# Longitudinal monitoring of CA125 levels provides additional information about survival in ovarian cancer

**DOI:** 10.1186/1757-2215-3-22

**Published:** 2010-10-12

**Authors:** Digant Gupta, Carolyn A Lammersfeld, Pankaj G Vashi, Donald P Braun

**Affiliations:** 1 Cancer Treatment Centers of America® at Midwestern Regional Medical Center, Zion, IL, 60099, USA

## Abstract

**Background:**

We investigated the prognostic impact of changes in serum CA125 levels during the first 3 months of therapy in ovarian cancer.

**Methods:**

A case series of 170 ovarian cancer patients treated at Cancer Treatment Centers of America. Based on CA125 levels at baseline and 3 months, patients were classified into 4 groups: 1) Normal (0-35 U/ml) at baseline and three months; 2) High (>35 U/ml) at baseline, normal at three months; 3) Normal at baseline, high at 3 months; 4) High at baseline and three months. Kaplan Meier method was used to calculate survival across the 4 categories.

**Results:**

Of 170 patients, 36 were newly diagnosed while 134 had received prior treatment. 25 had stage I disease at diagnosis, 15 stage II, 106 stage III and 14 stage IV. The median age at presentation was 54.2 years (range 23.1 - 82.5 years). At baseline, 31 patients had normal (0-35 U/ml) serum CA125 levels while 139 had high (>35 U/ml) levels. At 3 months, 59 had normal while 111 had high levels. Patients with a reduced CA125 at 3 months had a significantly better survival than those with increased CA125 at 3 months. Patients with normal values of CA125 at both baseline and 3 months had the best overall survival.

**Conclusions:**

These data show that reduction in CA125 after 3 months of therapy is associated with better overall survival in ovarian cancer. Patients without a significant decline in CA125 after 3 months of therapy have a particularly poor prognosis.

## Background

Ovarian cancer is the second most common gynecologic malignancy in the United States, with approximately 22,200 new cases each year [1]. It is also the leading cause of death from gynecologic cancers in the United States [2]. The overall lifetime risk of developing ovarian cancer for women in the United States is 1.4% to 1.8%. This risk varies from 0.6% for women with no family history, at least three term pregnancies, and four or more years of oral contraceptive use, to 3.4% for nulliparous women with no oral contraceptive use. For women with a family history, the lifetime risk for ovarian cancer is estimated at 9.4% [3].

Ovarian cancer is often asymptomatic in its early stages and thus most patients have widespread disease at the time of diagnosis [4]. Despite the achievements of high response rates with surgery followed by chemotherapy [5,6], 75% of women ultimately die of complications associated with disease progression. Although studies show that the survival of early-stage disease is significantly higher than those with advanced cancers, approximately 20% to 30% of these patients will die of their disease [7-9]. While the 5-year survival for women presenting with early-stage disease is approximately 90%, the majority of women (75%) are diagnosed with late stage disease (stage III or stage IV) and have a 5-year survival of less than 30% [10]. Mortality might be reduced if the disease is detected in the early stages [11].

The need for the development of reliable serum biomarkers for early detection and prognostication of ovarian cancer, which are both sensitive and specific, remains a long awaited priority. Investigators are aware of this need and the Early Detection Research Network (EDRN) established by the National Cancer Institute has proposed 'guidelines' for the development of screening biomarkers [12]. Over the last few decades a variety of serological tumor markers have been proposed as a supplement to other non-invasive diagnostic methods [13]. A variety of biomarkers have been developed which have the capacity to improve the dismal survival rate by monitoring growth of ovarian cancer and by detecting disease earlier. In the management of ovarian cancer these biomarkers have been applied for distinguishing malignant from benign pelvic masses, for monitoring response to treatment, for estimating prognosis, for predicting response to individual drugs, and for detecting primary disease at an early stage [14]. The most widely used marker of ovarian cancer, often considered the 'gold standard' is CA125 [15].

The role played by CA125 has developed over the past two decades, and presently CA125 remains the only tumor marker that has any significant impact on the clinical management of epithelial ovarian cancer [15]. Data from several studies have demonstrated a relationship between pre-chemotherapy serum CA125 levels [16-18] as well as post-chemotherapy serum CA125 levels and survival in women with epithelial ovarian malignancies [4,19-21]. Also, the relationship between CA125 levels and survival during chemotherapy has been evaluated in some studies [22,23]. Furthermore, the response to treatment and the clinical outcome of patients with epithelial ovarian cancer has been related to different parameters of evaluation of serum CA125 kinetics during early chemotherapy, such as the nadir CA125 level [24], the time to reach nadir level [25,26], and the most widely investigated kinetic parameter the serum CA125 half-life [22,26,27]. Similarly, some studies have evaluated the usefulness of the CA-125 area under the curve (AUC) as a new kinetic parameter for predicting overall survival in patients with ovarian cancer [28,29]. Finally, studies have evaluated the prognostic significance of pre-operative [18,30-32] as well as post-operative serum CA125 levels in ovarian cancer [18,31,33,34].

While there are numerous studies evaluating the relationship between CA125 assessment at a single time point and survival as mentioned above, only a few longitudinal studies have been carried out to this effect [35,36]. Those studies found a strong independent prognostic effect of a reduction in CA125 level during treatment, a change in CA125 level after the first course of chemotherapy, and CA125 half-life and nadir concentrations. We carried out the present study with the goal of further investigating the impact of serial improvement in CA125 levels on survival in patients with ovarian cancer.

## Methods

### Study Sample

A retrospective chart review was performed on a consecutive case series of 170 ovarian cancer patients treated at Cancer Treatment Centers of America^® ^(CTCA) at Midwestern Regional Medical Center (MRMC) between January 01 and May 06. None of these patients had received any treatment at MRMC prior to being enrolled in this investigation. The patients were identified from the MRMC tumor registry. All patients had histologically confirmed diagnosis of ovarian cancer. All patients received the same standard of care using an integrative model combining surgery, radiation and chemotherapy as appropriate, plus complementary therapy consisting primarily of nutritional, psychosocial, and spiritual support, naturopathic supplements, pain management, and physical therapy/rehabilitation.

### Covariates

Covariates that were assessed for prognostic significance were age at presentation, stage of disease at diagnosis and prior treatment history. The prior treatment history variable categorized patients into those who had received definitive cancer treatment elsewhere before coming to our institution and those who were newly diagnosed at our institution. The only follow-up information required was the date of death or the date of last contact/last known to be alive. This study was approved by the Institutional Review Board at MRMC.

### Data Analysis and Statistical Methods

All data were analyzed using SPSS 11.5 (SPSS Inc., Chicago, IL, USA). Based on their CA125 assessment at baseline (study entry) and 3 months, patients were classified into 4 groups of CA125 change: 1) Normal (0-35 U/ml) at baseline and three months; 2) High (>35 U/ml) at baseline, normal at three months; 3) Normal at baseline, high at 3 months; 4) High at baseline and three months. These 4 categories of CA125 change were compared with each other with respect to age at presentation, stage at diagnosis and prior treatment history using Chi-Square test or ANOVA as appropriate.

The Kaplan-Meier or product-limit method was used to calculate survival. The log rank test statistic was used to evaluate the equality of survival distributions across different strata. A difference was considered to be statistically significant if the p value was less than or equal to 0.05. For the purpose of evaluating the prognostic significance of CA125 at baseline, patient survival was defined as the time interval between date of first patient visit to the hospital and date of death from any cause or date of last contact/last known to be alive. While for the purpose of evaluating the prognostic significance of CA125 at 3 months, patient survival was defined as the time interval between date of patient visit at 3 months from first visit and date of death from any cause or date of last contact/last known to be alive. Survival was also evaluated using multivariate Cox regression analysis after adjusting for age at presentation, prior treatment history, and stage at diagnosis. Cox regression with time-invariant covariates assumes that the ratio of hazards for any two groups remains constant in proportion over time. We checked this assumption by examining log-minus-log plots for categorical predictors. Log-minus-logs plots showed that the assumptions were met for all three categorical predictors.

## Results

At the time of this analysis (June 08), 82 patients had expired and 88 were censored, as shown in Table 1. The cut-off date for the follow-up for all participants was June 08. The median age at presentation was 54.2 years (range 23.1 - 82.5 years). At baseline (study entry), 31 patients had normal serum CA125 levels (0-35 U/ml) and 139 had high serum CA125 levels (>35 U/ml). At 3 months, 59 patients had normal serum CA125 levels (0-35 U/ml) and 111 had high serum CA125 levels (>35 U/ml). The median serum CA125 levels at baseline and 3 months were 152 U/ml (range: 5 - 16200 U/ml) and 108.5 U/ml (range: 3 - 15800 U/ml) respectively.

**Table 1:** Patient Characteristics

Characteristic	Categories	Number	Percent (%)
Vital Status	Expired	82	48.2
	Censored^*1*^	88	51.8
Prior Treatment	Previously treated disease	134	78.8
History	Newly diagnosed	36	21.2
Stage at Diagnosis	Stage I	25	14.7
	Stage II	15	8.8
	Stage III	106	62.4
	Stage IV	14	8.2
	Missing	10	5.9
Age at Presentation	Mean	53.4	
	Median	54.2	
	Range	23.1 - 82.5	
Baseline CA125 at study entry	0-35 U/ml	31	18.2
	>35 U/ml	139	81.8

Of 35 newly diagnosed patients, 24 (68.6%) had stage III or IV disease while 96 (76.8%) of 125 previously treated patients had stage III or IV disease, the difference being statistically significant (p = 0.03). At baseline, 10 of 36 (27.8%) newly diagnosed patients had normal CA125 levels, while 21 of 134 (15.7%) of previously treated patients had normal CA125 levels, the difference being statistically non-significant (p = 0.09). At baseline, 12 of 40 (30%) early-stage (stage I and II) patients had normal CA125 levels while 19 of 120 (15.8%) late-stage (stage III and IV) patients had normal CA125 levels, the difference being statistically significant (p = 0.04) suggesting that patients with advanced stage disease had higher CA125 levels.

**Figure 1:**
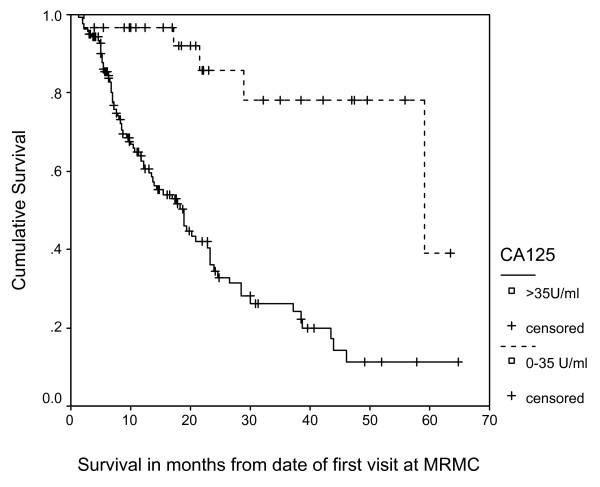
**Survival Curves for 2 Categories of CA125 at Baseline**.

**Figure 2:**
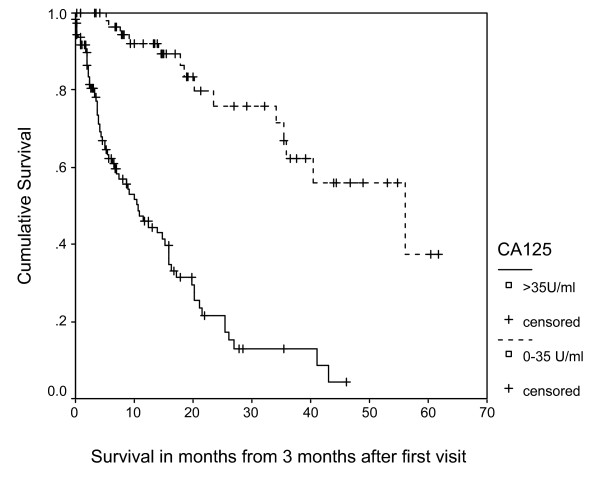
**Survival Curves for 2 Categories of CA125 at 3 Months**.

Figure 1 displays the Kaplan-Meier survival curves for the 2 categories of serum CA125 at baseline. The median survival for patients with normal CA125 (N = 31) was 59.2 months while for those with high CA125 (N = 139) was 18.8 months (log rank = 20.1, p < 0.0001). Figure 2 displays the Kaplan-Meier survival curves for the 2 categories of serum CA125 at 3 months. The median survival for patients with normal CA125 (N = 59) was 56.2 months while for those with high CA125 (N = 111) was 10.7 months (log rank = 44.6, p < 0.0001).

**Figure 3:**
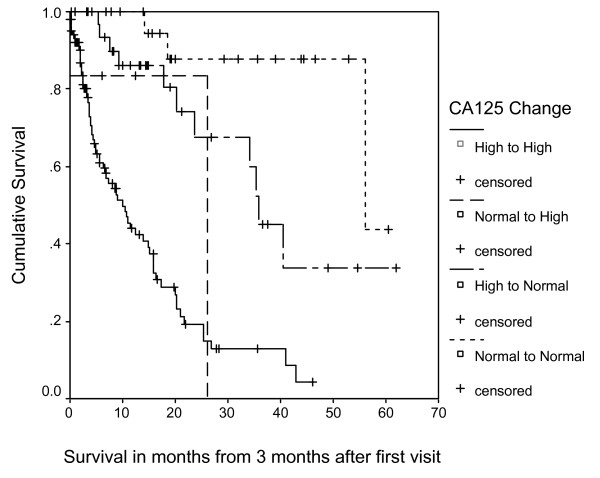
**Survival Curves for 4 Categories of CA125 Change**.

Table 2 describes the median survival (Kaplan-Meier) for the 4 classes of people based on their CA125 scores at baseline and 3 months. Figure 3 displays the survival curves for the 4 classes of change in serum CA125. In this cohort, patients with an improved (decreased) serum CA125 levels at 3 months (stratum 2) had a significantly better survival than those with deteriorated (increased) serum CA125 levels at 3 months (stratum 3). Patients with normal serum CA125 levels after 3 months (strata 1 and 2) had increased survival compared to patients with high serum CA125 levels at 3 months (strata 3 and 4).

**Table 2:** Median Survival for 4 Categories of CA125 Change

Strata	Baseline CA125 at study entry	3 month CA125	N	Median Survival (in months)	95% CI	Kaplan Meier Log-Rank P-value
**1**	Normal	Normal	25	56.1	3.5-108.8	
**2**	High	Normal	34	35.8	33.1-38.4	
**3**	Normal	High	6	21.7	10.8-32.6	<0.0001
**4**	High	High	105	10.1	6.2-13.9	

Normal = 0-35 U/ml

High = >35 U/ml

Table 3 describes the comparison between 4 categories of CA125 change with respect to age at presentation, stage at diagnosis and prior treatment history using Chi-Square test and ANOVA as appropriate. Seventeen of 34 (50%) patients who changed from high at baseline to normal at 3 months (stratum 2) had previously treated disease, whereas 96 of 105 (91.4%) patients who had high CA125 levels at both baseline and 3 months had previously treated disease, the difference being statistically significant. In other words, patients in stratum 4 (high to high) had a greater percentage of patients with previously treated disease. This finding partly explains why patients in stratum 4 who had high CA125 levels at baseline (N = 105) failed to achieve an improved CA125 level at 3 months. Similarly, a greater percentage of patients in stratum 4 had advanced stage disease as compared to those in stratum 2. Age at presentation did not vary across the 4 categories of CA125 change.

**Table 3:** Relationship between Change in CA125 and Covariates

Variable	Change in CA125 from Baseline to 3 months N (%)				P
	Normal to Normal	High to Normal	Normal to High	High to High	
Prior Treatment History					
• Previously treated disease	15 (60)	17 (50)	6 (100)	96 (91.4)	**<0.001 **(chi-square)
• Newly diagnosed	10 (40)	17 (50)	0 (0)	9 (8.6)	
Stage at Diagnosis					
• Stage I	10 (40)	4 (14.3)	0 (0)	11 (10.9)	
• Stage II	2 (8)	6 (21.4)	0 (0)	7 (6.9)	**0.009 **(chi- square)
• Stage III	11 (44)	15 (53.6)	5 (83.3)	75 (74.3)	
• Stage IV	2 (8)	3 (10.7)	1 (16.7)	8 (7.9)	
	**Mean (Standard Deviation)**				
Age at presentation	52.3 (12.9)	55.4 (11.5)	54.1 (6.3)	52.9 (10.2)	0.67 (ANOVA)

Table 4 describes the results of multivariate Cox regression modeling. Multivariate time-independent Cox modeling, after adjusting for age at presentation, stage at diagnosis and prior treatment history found that change in CA125 from high to normal was associated with a protective relative risk of 0.29 as compared to no change in high CA125 status from baseline to 3 months. Similarly, maintenance of normal CA125 status from baseline to 3 months was associated with a protective relative risk of 0.07 as compared to no change in high CA125 status from baseline to 3 months. Age at presentation, stage at diagnosis and prior treatment history were not found to be statistically significantly associated with survival as shown in Table 4. The overall model was statistically significant (p < 0.001).

**Table 4:** Multivariate Cox Proportional Hazard Model

Independent Variable	Unit of increase	**RR**^***1***^	95% CI	P-value
Age at presentation	1 year	1.003	0.98, 1.03	0.78
Prior treatment history	Newly diagnosed as referent	1.1	0.52, 2.2	0.86
Stage at Diagnosis				
• Stage II		0.29	0.06, 1.5	0.15
• Stage III	Stage I as referent	1.4	0.61, 3.2	0.43
• Stage IV		1.7	0.57, 5.0	0.35
Change in CA125				
• Normal to Normal		0.07	0.02, 0.26	**<0.001**
• High to Normal	High to High as referent	0.29	0.13, 0.65	**0.003**
• Normal to High		0.40	0.09, 1.7	0.21

^*1 *^Relative risk (Cox proportional hazard)

N = 160

## Discussion

CA125 is considered the 'gold standard' tumor marker in ovarian cancer and has a significant impact on the clinical management of the disease such as monitoring of treatment response and disease progression. Several studies have evaluated the prognostic role of CA125 assessments at various time points during cancer treatment. However, there is little to no data in the literature documenting the impact of serial measurements of CA125 on survival in ovarian cancer. As a result, the current study was undertaken to address this important research question. We found that patients with an improved CA125 levels 3 months had a significantly better survival than those with deteriorated CA125 levels at 3 months. We also found that 3 month CA125 was a better predictor of survival as compared to baseline CA125. These observations suggest that a patient's CA125 levels at 3 months after treatment might have greater clinical significance as compared to a patient's CA125 levels at presentation for treatment.

In order to put our study in better context within the existing literature, we review here two similar studies which have examined the relationship between longitudinal assessment of CA125 and survival in ovarian cancer. A study by Markman M. et al. investigated the relationship between early changes in serum CA125 and survival in patients with advanced ovarian cancer. The serum CA125 values from 101 patients with advanced ovarian cancer who participated in a Southwest Oncology Group trial (SWOG 8412) were evaluated. All patients had CA 125 values available for at least 8 weeks following initiation of chemotherapy. While pre-treatment CA125 values did not correlate with survival, the concentration of this tumor marker 8 weeks after initiation of therapy was a powerful independent prognostic factor. The median survivals for patients (n = 51) with a CA125 <35 U/ml, vs. patients (n = 50) with a CA125 >35 U/ml, at this time point, were 26 months and 15 months, respectively (P = 0.0001). Furthermore, women with serum CA125 values <50% of their pre-treatment concentration at 8 weeks experienced a median survival of 21 months, compared to only 10 months for individuals with tumor marker levels >50% of their baseline value (P = 0.0003). The study concluded that the reduction in the serum CA125 concentration over the initial two cycles of platinum-based chemotherapy is a powerful independent predictor of survival for patients with suboptimal stage III or IV ovarian cancer [35].

Another study by Riedinger JM et al. assessed the prognostic value of the CA125 change after the first and the second courses of induction chemotherapy in 494 patients with epithelial ovarian cancer. CA125 determination of all patients was carried out before each cycle of chemotherapy (on average 3 weeks) and different biological variables derived from CA125 kinetics during the first two chemotherapy courses were also examined. Changes in CA125 were classified into six groups. The data from the study showed that early CA125 change during the first chemotherapy course and CA125 before the second chemotherapy course were strongly correlated with survival [36].

The findings of our study, specifically the positive impact of CA125 reduction on overall survival, compare well with those of the above mentioned studies. The key difference between our study and those mentioned above revolves around the method of calculating CA125 improvements or deterioration over time. While we used the cut-off of 35 U/ml to divide our patient population into 4 groups based on their CA125 levels at baseline and 3 months, the other 2 studies used percentage change methodology to quantify the increase as well as decrease in CA125 levels over time. We investigated the predictive value of CA125 normalization in addition to evaluating the absolute levels at baseline and 3 months after treatment. While the SWOG 8412 study by Markman M. et al. included only patients with suboptimal residual stage III (defined as the presence of at least one tumor mass >2 cm in diameter remaining within the peritoneal cavity following initial cytoreductive surgery) or IV disease and the study by Riedinger JM et al. included patients with stages IIc-IV disease only, our study included patients across all disease stages (I-IV). Therefore, our study adds useful information to the growing body of literature on the impact of CA125 improvements on survival during treatment in patients with ovarian cancer.

Some limitations of this study require acknowledgment. Our study, because of its retrospective nature, relies on data not primarily meant for research. In order to compare CA125 response to treatment and correlate it with survival, a homogeneous group of patients must be evaluated. However, we evaluated a mixed population of patients (newly diagnosed as well those who have been treated previously). We think that restricting the analysis to newly diagnosed patients (patients with no prior treatment history) would have been more accurate, since it would have allowed for evaluation of true overall survival time i.e. time from the date of diagnosis to the date of death. Specifically, survival time for previously treated patients in our study is longer than measured because of the time between their initial diagnosis and presentation at CTCA (this primarily affected the estimated survival of patients in stratum 4, since over 90% of these patients had previously treated disease). However, doing so would have caused a significant reduction in the sample size. Moreover, the prior treatment history variable was adjusted for in the multivariate analysis. In our study, the survival time was calculated from the day of first visit at our hospital because information on CA125 was not always available at the time of diagnosis for previously treated patients. This drawback emphasizes the need for conducting prospective studies having CA125 information available since the date of diagnosis. A majority of our patients had advanced stage disease and had failed primary treatment elsewhere before coming to our hospital. As a result, generalizability of the study findings to cancer patients with early-stage disease might be questionable. However, we have no reasons to believe that patients with early-stage disease will display different findings. Because of the retrospective nature of our study and lack of information on treatments received by our patients, the specific implications of our findings on managing therapy in ovarian cancer patients are uncertain. Prospective studies are needed to answer this specific question.

The utility of serum CA125 measurements in managing therapy in ovarian cancer patients continues to be a subject of great research interest. A randomized trial was designed by Rustin GJ. et al. to determine whether there were benefits from early treatment based on a confirmed elevation of CA125 levels versus delaying treatment until clinically indicated. Patients with complete remission after first-line platinum-based chemotherapy whose CA125 levels exceeded twice the upper limit of normal were randomized to either immediate treatment or to remain having blinded CA125 measurements with treatment commencing when clinical or symptomatic recurrence appeared. No survival benefit from early treatment based on a raised serum marker level alone was found, and therefore it was concluded that there is no value in the routine measurement of CA125 in the follow-up of ovarian cancer patients [37]. Our study, on the other hand, confirms that both baseline and short term (3 months) improvement in CA125 levels is associated with better long term survival in ovarian cancer. Thus, the CA125 trend seems to be a useful prognostic predictor at different time points along the disease trajectory for both newly diagnosed as well as previously treated patients. While this is no news to most clinicians, the information about length of survival as a function of CA125 assessment might be helpful to clinicians trying to counsel ovarian cancer patients about prognosis, especially for patients who are well into the course of their disease. Consequently, our findings lend support to the importance of regular monitoring of CA125 during the entire spectrum of treatment in ovarian cancer from the point of view of predicting survival.

## Competing interests

The authors declare that they have no competing interests.

## Authors' contributions

DG and CAL participated in concept, design, data collection, data analysis, data interpretation and writing. PGV and DBP participated in concept, design and data interpretation. All authors read and approved the final manuscript.
